# The lncRNA ALMS1‐IT1 may promote malignant progression of lung adenocarcinoma via AVL9‐mediated activation of the cyclin‐dependent kinase pathway

**DOI:** 10.1002/2211-5463.13140

**Published:** 2021-04-03

**Authors:** Tian Luan, Tian‐Ye Zhang, Zhong‐Hua Lv, Bi‐Xi Guan, Jian‐Yu Xu, Jian Li, Ming‐Xu Li, Song‐Liu Hu

**Affiliations:** ^1^ Department of Radiation Oncology Harbin Medical University Cancer Hospital Harbin China

**Keywords:** ALMS1‐IT1, AVL9, CDK pathway, lung adenocarcinoma, prognosis

## Abstract

Lung adenocarcinoma (LUAD) is the primary epithelial tumor of the lung. The lack of clinical symptoms and specific molecular diagnostic indicators during the early stages of LUAD mean that the disease may not be detected until late stages, and the 5‐year survival rate is only approximately 15%. Long non‐coding RNA ALMS1 intronic script 1 (ALMS1‐IT1) was previously reported to be correlated with the poor prognosis of head and neck squamous cell carcinoma patients. Here, we investigated whether ALMS1‐IT1 has prognostic potential for LUAD. Bioinformatics analyses were performed to examine the expression and prognostic value of ALMS1 and AVL9 (for which gene expression is positively correlated with ALMS1‐IT1 expression in LUAD) in LUAD based on TCGA and Oncomine databases. We report that ALMS1‐IT1 and AVL9 were both highly expressed in LUAD and correlated with poor outcomes in LUAD patients. Of note, the prognosis of LUAD patients with low expression of both ALMS1‐IT1 and AVL9 was superior to that of other patients. Furthermore, the proliferation, migration and invasion of LUAD cells were decreased in cells lacking ALMS1‐IT1, and this decrease could be almost completely reversed through overexpression of AVL9. Gene set enrichment analysis revealed that expression of genes related to the cell cycle pathway is closely related to both the high expression of ALMS1‐IT1 and AVL9 in LUAD. Finally, up‐regulation of ALMS1‐IT1 can activate the cyclin‐dependent kinase pathway, whereas absence of AVL9 can reverse this activation, as shown by western blotting. In summary, ALMS1‐IT1/AVL9 may promote the malignant progression of LUAD, at least in part by regulating the cyclin‐dependent kinase pathway.

## Graphical abstract

Lung adenocarcinoma (LUAD) is the primary epithelial tumor of the lung. We report here that long non‐coding RNA ALMS1 intronic script 1 (ALMS1‐IT1) and AVL9 are both highly expressed in LUAD and correlated with poor outcomes in LUAD patients. ALMS1‐IT1/AVL9 may promote the malignant progression of LUAD, at least in part by regulating the cyclin‐dependent kinase pathway.

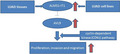

**Abbreviations**
ALMS1‐IT1: ALMS1 intronic script 1
CCK‐8: Cell Counting Kit‐8
CDK: cyclin‐dependent kinase
GSEA: gene set enrichment analysis
HNSCC: head and neck squamous cell carcinoma
lncRNA: long non‐coding RNA
LUAD: lung adenocarcinoma
ncRNAs: non‐coding RNAs
qRT‐PCR: quantitative real‐time PCR

Lung adenocarcinoma (LUAD) is the primary epithelial tumor of the lung, mostly originating from the epithelium of bronchial mucosa or alveolar epithelium [1]. Recently, the incidence rate of LUAD has been increasing and has become the most common type of lung cancer worldwide [2, 3]. Because of a lack of clinical symptoms and specific molecular diagnostic indicators in the early stage of LUAD, patients are likely to miss the best opportunity for treatment, and the 5‐year survival rate is only approximately 15% [4]. With the rapid development of medical technology, proteomics has been widely applied, and precision medicine has been gradually developed [5]. Hence, the selection of reliable biomarkers has great clinical significance for improving the accuracy of diagnosis and formulating effective treatment strategies in LUAD.

Non‐coding RNAs (ncRNAs) are considered as types of transcript that do not encode any protein [6] and are involved in the regulation of many biological processes [7, 8]. Based on the length of nucleotide sequence, ncRNAs are divided into short ncRNAs and long ncRNAs (lncRNAs) [9]. lncRNAs have more than 200 nucleotides, which can regulate transcription, chromatin modification and gene expression [10]. It is remarkable that the imbalance of lncRNAs is related to the progression and prognosis of cancers [11, 12], including LUAD [13]. In the present study, we found that ALMS1 intronic script 1 (ALMS1‐IT1) was dysregulated in LUAD based on bioinformatics analysis. ALMS1‐IT1, an lncRNA, has been recognized to have prognostic value [14]. Lu Xing *et al*. [15] reported that ALMS1‐IT1 was up‐regulated in the high‐risk group of head and neck squamous cell carcinoma (HNSCC) and this was related to the poor prognosis of HNSCC patients. Furthermore, it was also found that ALMS1‐IT1 was the lncRNA that targets the most miRNAs and proteins in HNSCC [15]. Hence, we speculate that ALMS1‐IT1 has great research value in LUAD, although the specific function of ALMS1‐IT1 in LUAD is unclear.

To the best of our knowledge, lncRNAs do not encode functional proteins and, instead, exert biological functions by influencing gene expression [16]. In the present study, bioinformatics analysis revealed that AVL9 and ALMS1‐IT1 had a significant co‐expression relationship. AVL9, a migration‐related protein, has been confirmed to participate in multiple biological processes, such as cell cycle and cell migration [17]. Importantly, it has been reported that the knockdown of AVL9 obviously restrained the migration of human adenocarcinoma alveolar basal epithelial cells A549 [17]. Nevertheless, the function of AVL9 in LUAD has not been determined.

In the present study, we investigated both the expression and biological functions of ALMS1‐IT1 in LUAD, and also considered whether ALMS1‐IT1 worked in coordination with AVL9 in the malignant progression of LUAD. The results are expected to provide feasible biomarkers for LUAD diagnosis and therapy.

## Materials and methods

### Bioinformatics analysis

Data were obtained from the TCGA database (https://www.cancer.gov/about‐nci/organization/ccg/research/structural‐genomics/tcga) to analyze the differential expression of ALMS1‐IT1 in LUAD tissues, which included 535 LUAD tissues and 55 paired adjacent normal lung tissues. We also analyzed the expression of AVL9 based on the mRNA expression data from the TCGA and Oncomine databases (https://www.cancer.gov/about‐nci/organization/ccg/research/structural‐genomics/tcga). Concurrently, Kaplan–Meier survival analysis was performed to determine the correlation between the expression of ALMS1‐IT1, AVL9, ALMS1‐IT1/AVL9 low or ALMS1‐IT1/AVL9 high and the outcomes of patients with LUAD using the data from the TCGA database. Furthermore, the correlation between ALMS1‐IT1 and AVL9 in LUAD was analyzed using Pearson correlation coefficient analysis.

In addition, gene set enrichment analysis (GSEA) was utilized to explore the pathway highly related to the high expression of ALMS1‐IT1 and AVL9 in LUAD. GSEA analysis was conducted with LUAD samples in the TCGA database (accession [TCGA‐LUAD]). We divided the samples into high‐ and low‐ expression groups according to the median expression of ALMS1‐IT1 and AVL9, and chose the C2 (c2.cp.kegg.v6.0.symbols.gmt) sub‐collection downloaded from the Molecular Signatures Database (http://software.broadinstitute.org/gsea/msigdb/index.jsp) as the reference gene sets for performing GSEA analysis.

### Cells culture and transfection

Human LUAD cell lines Calu‐3, NCI‐H1395, SPC‐A1 and NCI‐H2009, as well as normal cells BEAS2B and NHBE, were all ordered from American Type Culture Collection (Manassas, CA, USA). RPMI‐1640 medium, which supplemented with 10% fetal bovine serum (Gibco, Carlsbad, CA, USA), streptomycin (0.1 mg·mL^−1^) and penicillin (100 U·mL^−1^), was utilized to culture the cells at 37 °C with 5% CO_2_.

Calu‐3 and NCI‐H2009 cells were used for transfection. pcDNA3.1‐ALMS1‐IT1 and si‐ALMS1‐IT1 (5ʹ‐ TTGCGAAATAGTGTTTGTCTAGA‐3ʹ) were obtained from Shanghai GenePharma Co., Ltd (Shanghai, China) and applied for overexpression/knockdown of ALMS1‐IT1. Consistently, pcDNA3.1‐AVL9 and si‐AVL9 (5ʹ‐CCTCAGAGAGTCTTCCAATTACTC‐3ʹ) were also synthesised by Shanghai GenePharma Co., Ltd and used for overexpression/knockdown of AVL9. si‐con (5ʹ‐AGTGTTCATGAAGCCATGGATTC‐3ʹ) and vector were used as a control. Lipofectamine 2000 (Thermo Fisher Scientific, Waltham, MA, USA) was utilized for transfection in accordance with the manufacturer’s instructions. After 48 hours of transfection, a quantitative real‐time polymerase chain reaction (qRT‐PCR) and western blot were conducted to examine the transfection efficiency, and cells were collected for follow‐up experiments.

### Quantitative real‐time PCR

An RNeasy Mini kit (Qiagen, Valencia, CA, USA) was utilized to extract the total RNA from cells in accordance with the manufacturer’s instructions. Complementary DNA was gained using a reverse‐transcribed assay with SYBR Premix Ex Taq II (TaKaRa, Shiga, Japan) in accordance with the manufacturer’s instructions. Then, the expression of ALMS1‐IT1 and AVL9 was measured using qRT‐PCR analysis with SYBR Green I (Invitrogen) in accordance with the manufacturer’s instructions. The data were computed using the 2^−ΔΔCT^ method [18] and normalized to GAPDH. A qRT‐PCR was conducted with the forward primer (5ʹ‐GCAGTGGTTCTTGACGGGTA‐3’) and the reverse primer (5ʹ‐CAGTCCAGCCTGGGCAATAA‐3ʹ ) for ALMS1‐IT1, as well as the forward primer (5ʹ‐AGGATACCTGGGATGGCTGT‐3ʹ) and the reverse primer (5ʹ‐ACAGCCATCCCAGGTATCCT‐3ʹ) for AVL9. GAPDH (forward: 5ʹ‐CCATGGGGAAGGTGAAGGTC‐3ʹ, reverse: 5ʹ‐GACCTTCACCTTCCCCATGG‐3ʹ) was used as an internal control.

### Western blotting analysis

Ice‐cold RIPA Lysis Buffer (CWBIO, Beijing, China) was used to lyse the cells, and the proteins were extracted with M‐PER Mammalian® Protein Extraction Reagent (Thermo Fisher Scientific, USA). Then, the protein concentration was detected using a BCA Protein Assay Kit (Pierce, Rockford, IL, USA). Proteins (30 μg) were electrophoresed via SDS/PAGE, followed by transfer to a poly(vinylidene difluoride) membrane. After being sealed with 5% non‐fat milk for 1 h, the membranes were incubated with anti‐AVL9 (dilution 1 : 1000), anti‐CDK1 (dilution 1 : 1000), anti‐p‐CDK1 (dilution 1 : 1000), anti‐CDK2 (dilution 1 : 500), anti‐p‐CDK2 (dilution 1 : 1000), anti‐CCNE1 (dilution 1 : 1000), anti‐CCNB1 (dilution 1 : 1000) or anti‐GAPDH (dilution 1 : 1000) at 4 °C overnight. All primary antibodies were obtained from Biorbyt Ltd (Cambridge, UK). Subsequently, the membrane was washed three times for 5 min, which was then incubated with horseradish peroxidase‐conjugated secondary antibody (Santa Cruz Biotechnology, Inc., Santa Cruz, CA, USA) for 1 h at room temperature. After rinsing, an ECL Western Blotting Kit (Thermo Fisher Scientific) was used to visualize the blots. Finally, the gray value was scanned using quantity one (Bio‐Rad, Hercules, CA, USA) and the relative expression of each protein was calculated with GAPDH as an internal reference.

### Proliferation assay

Cell viability and proliferation were measured using Cell Counting Kit‐8 (CCK‐8) and colony formation assays [19]. For the CCK‐8 assay, the cultured cells were digested and used to prepare cell suspension. Cells were inoculated in a 96‐well plate (1000 cells per well) and routinely cultured with 5% CO_2_. Cell viability was measured at different time points (0, 24, 48 and 72 h). Of note, 10 μL of CCK‐8 solution was added to each well before detection and then incubated for 1.5 h at 37 °C. The optical density was calculated using a microplate reader at a wavelength of 450 nm. The attenuance values were used to construct the multiplication curve.

For colony formation assay, approximately 500 treated cells were inoculated in each medium to culture for 7–14 days with 5% CO_2_ at 37 °C and saturated humidity until cells formed naked‐eye‐visible colonies. The cells were fixed utilizing 80% ethanol for 30 min, and then the fixative was removed, followed by staining with 0.1% crystal violet for 30 min. Finally, the number of colonies was counted.

### Migration and invasion assays

A transwell assay was carried out to assess the invasion and migration of transfected cells [20]. Briefly, a transwell chamber pre‐coated with Matrigel matrix glue (100 μL; serum‐free medium diluted 1:6; BD Biosciences, San Jose, CA, USA) was utilized for the invasion assay. Cells were re‐suspended with serum‐free culture medium, followed by the addition of approximately 1 × 10^5^ cells to the upper chamber. Concurrently, the lower chamber was added the complete medium as the chemical attractant. Following cultivation for 24 h, non‐invading cells were erased, and cells that invaded through the membrane were fixed with 4% paraformaldehyde for 30 min and then stained with the Diff‐Quik stain (Sysmex, Kobe, Japan) for 30 min. After cleaning with PBS, the stained cells were counted. The migration assay was performed using a method similar to that for the invasion assay except, for a lack of Matrigel matrix glue.

### Statistical analysis

The experimental data were analyzed using spss, version 22.0 (IBM Corp., Armonk, NY, USA). Differences were compared using *t*‐test for two groups and one‐way analysis of variance with Tukey’s post‐hic test for multiple groups. Survival curves were plotted by Kaplan–Meier survival analysis, and the high‐expression group and the low‐expression groups were grouped based on the median expression level of ALMS1‐IT1/AVL9. Cox regression analysis was conducted to confirm whether ALMS1‐IT1/AVL9 could be used as an independent predictive factor of the prognosis of LUAD. *P* < 0.05 was considered statistically significant.

## Results

### ALMS1‐IT1 was expressed at high levels in LUAD and closely related to the poor outcomes

**Fig. 1:**
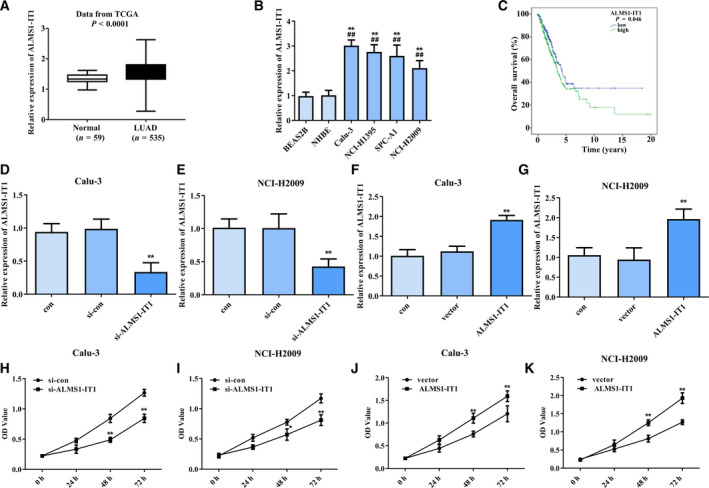
ALMS1‐IT1 expression in LUAD tissues and cell lines. (A) ALMS1‐IT1 expression in LUAD tissues (*n* = 535) and normal control tissues (*n* = 59) based on the TCGA database. *P* < 0.0001. (B) ALMS1‐IT1 expression in LUAD cell lines Calu‐3, NCI‐H1395, SPC‐A1 and NCI‐H2009 was measured using qRT‐PCR. **P < 0.01 vs. BEAS2B cell, ##*P* < 0.01 vs. NHBE cell. (C) Kaplan–Meier analysis was used to assess the relationship between the ALMS1‐IT1 level and survival times based on TCGA database. *P* < 0.05. (D, E) ALMS1‐IT1 expression in Calu‐3 cells and NCI‐H2009 cells transfected with si‐ALMS1‐IT1 or si‐con was detected by qRT‐PCR. **P < 0.01 vs. si‐con group. (F, G) ALMS1‐IT1 expression in Calu‐3 cells and NCI‐H2009 cells transfected with pcDNA3.1‐ALMS1‐IT1 or vector was detected by qRT‐PCR. **P < 0.01 vs. vector group. (H, I) The viability of Calu‐3 cells and NCI‐H2009 cells transfected with si‐ALMS1‐IT1 or si‐con was detected using a CCK‐8 assay. **P < 0.01 vs. si‐con group. (J, K) The viability of Calu‐3 cells and NCI‐H2009 cells transfected with pcDNA3.1‐ALMS1‐IT1 or vector was detected using a CCK‐8 assay. **P < 0.01 vs. vector group. Error bar represents the mean ± SD derived from three independent experiments. Comparisons between groups were analyzed using *t‐*tests (two‐sided).

First, ALMS1‐IT1 expression was assessed in 535 LUAD tissues and 55 paired adjacent normal lung tissues using bioinformatics analysis. As shown in Fig. 1A, ALMS1‐IT1 was up‐regulated in LUAD tissues compared to adjacent normal lung tissues (*P* < 0.0001). Then, we conducted a qRT‐PCR to confirm the ALMS1‐IT1 level in LUAD cell lines. As expected, ALMS1‐IT1 was also highly expressed in LUAD cell lines Calu‐3, NCI‐H1395, SPC‐A1 and NCI‐H2009 (*P* < 0.01) (Fig. 1B). Moreover, the data from Kaplan–Meier survival analysis indicated that patients with high ALMS1‐IT1 expression presented a shorter survival time (*P* = 0.046) (Fig. 1C). Cox regression analysis revealed that ALMS1‐IT1 can be used as prognostic factor but not as an independent predictor in LUAD patients (Table 1). These findings revealed that the malignant transformation of LUAD resulted in an increase in ALMS1‐IT1 expression.

**Table 1:** ALMS1‐IT1 can be used as a prognostic factor for lung adenocarcinoma based on the TCGA database.

Variables	Univariate analysis			Multivariate analysis		
	*P* value	HR	95% CI	*P* value	HR	95% CI
ALMS1‐IT1 expression	0.047*	1.358	1.004–1.838	0.057	1.343	0.991–1.819
Stage	0.000*	2.368	1.721–3.256	0.541	1.149	0.736–1.796
Pathologic‐T	0.000*	2.223	1.516–3.260	0.004*	1.852	1.213–2.829
Pathologic‐M	0.022*	1.943	1.103–3.426	0.278	1.418	0.754–2.667
Pathologic‐N	0.000*	2.471	1.830–3.335	0.000*	2.170	1.519–3.100
Age	0.694	1.070	0.763–1.500			
Gender	0.544	1.097	0.814–1.479			

CI, confidence interval; HR, hazard ratio.

*P < 0.05.

### Overexpression of ALMS1‐IT1 promoted the viability of LUAD cells

Next, NCI‐H2009 and Calu‐3 cells were transfected with pcDNA3.1‐ALMS1‐IT1, si‐ALMS1‐IT1 or the corresponding control and knockdown/overexpression transfection efficiency was detected. As shown in Fig. 1D,E, Calu‐3 cells and NCI‐H2009 cells transfected with si‐ALMS1‐IT1 resulted in a remarkable reduction in ALMS1‐IT1 expression, whereas pcDNA3.1‐ALMS1‐IT1 can enhance the ALMS1‐IT1 expression in both Calu‐3 cells and NCI‐H2009 cells compared to the control and vector groups (*P* < 0.01) (Fig. 1F,G).

Then, a CCK‐8 assay was conducted to assess the impact of ALMS1‐IT1 on cell viability. Our data indicated that knockdown of ALMS1‐IT1 markedly restrained the viability of Calu‐3 cells and NCI‐H2009 cells (*P* < 0.01) (Fig. 1H,I), whereas overexpression of ALMS1‐IT1 obviously enhanced the viability of Calu‐3 cells and NCI‐H2009 cells (*P* < 0.01) (Fig. 1J,K). These results established a causal relationship between ALMS1‐IT1 level and LUAD cell viability, with ALMS1‐IT1 contributing to promoting the viability of LUAD cells.

### AVL9 was positively correlated with ALMS1‐IT1 expression in LUAD

**Fig. 2:**
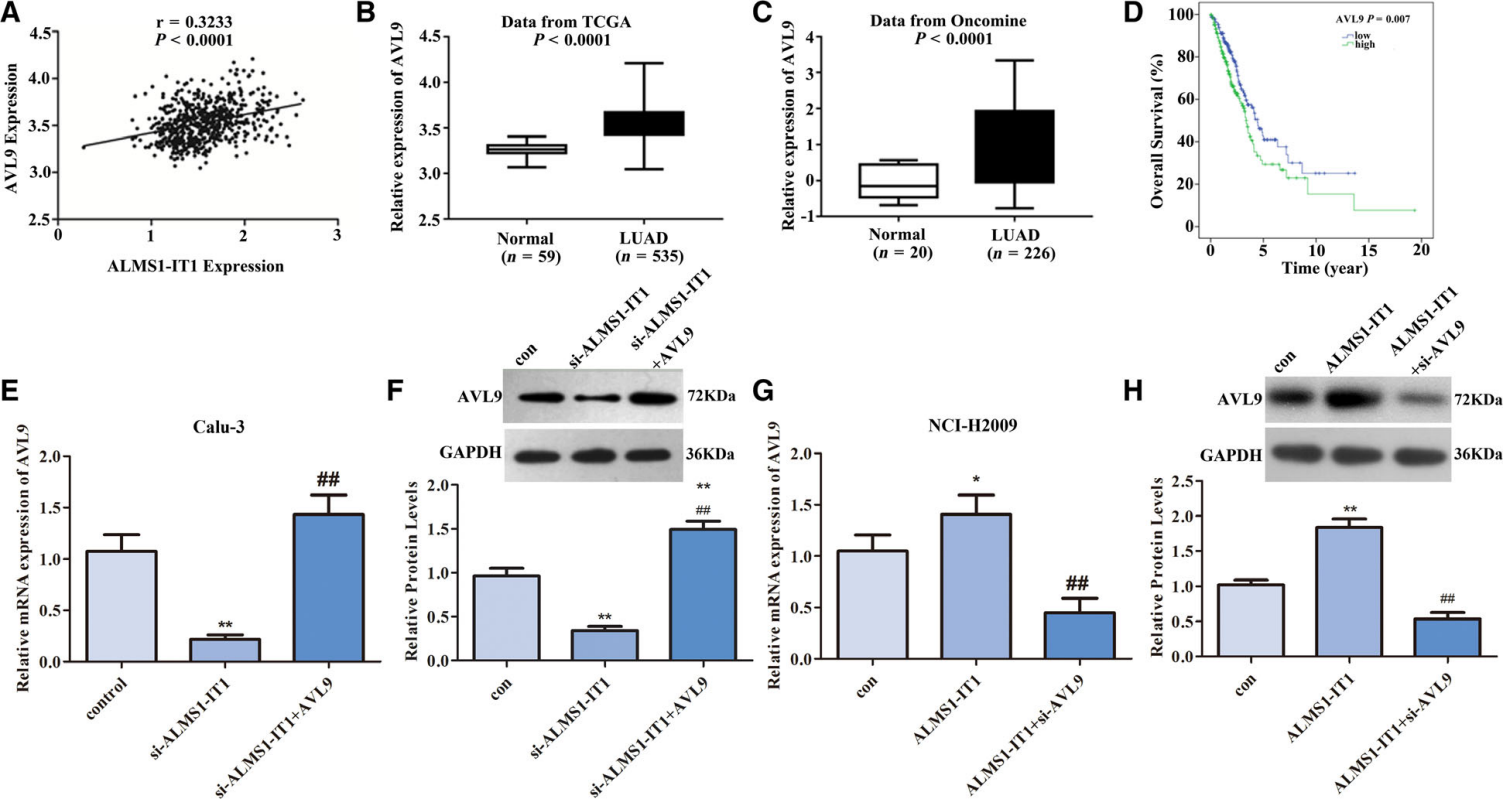
The relationship between ALMS1‐IT1 and AVL9 in LUAD. (A) Pearson’s correlation coefficient analysis was used to detect the correlation between ALMS1‐IT1 and AVL9 in LUAD. (B) AVL9 expression in LUAD tissues (*n* = 535) and adjacent normal lung tissue (*n* = 59) based on the TCGA database. *P* < 0.0001. (C) AVL9 expression in LUAD tissues (*n* = 226) and normal control tissues (*n* = 20) based on the Oncomine database. *P* < 0.0001. (D) The relationship between the AVL9 level and survival time was analyzed using Kaplan–Meier analysis, according to the TCGA database. *P* < 0.01. (E) mRNA and (F) protein levels of AVL9 in Calu‐3 cells transfected with si‐ALMS1‐IT1 or si‐ALMS1‐IT1 + pcDNA3.1‐AVL9 were measured using qRT‐PCR and western blotting. **P < 0.01 vs. control group, ##*P* < 0.01 vs. si‐ALMS1‐IT1 group. (G) mRNA and (H) protein levels of AVL9 in NCI‐H2009 cells transfected with pcDNA3.1‐ALMS1‐IT1 or pcDNA3.1‐ALMS1‐IT1 + si‐AVL9 were measured using qRT‐PCR and western blotting. **P < 0.01 vs. control group, ##*P* < 0.01 vs. pcDNA3.1‐ALMS1‐IT1 group. Error bar represents the mean ± SD derived from three independent experiments. Comparisons between groups were analyzed using *t‐*tests (two‐sided).

In view of the above results, we further investigated how ALMS1‐IT1 functioned in LUAD. Pearson correlation coefficient analysis was performed to analyze mRNA co‐expressed with ALMS1‐IT1. Linear regression analysis indicated that there were 268 differential expressed genes with absolute correlation coefficient ≥ .3. As shown in Fig. 2A, AVL9 was positively correlated with ALMS1‐IT1 expression (*P* < 0.0001) (*r* = 0.3233). In addition, because it has been reported that knockdown of AVL9 significantly restrained the migration of A549 cells [21], AVL9 was selected as the mRNA that co‐expressed with ALMS1‐IT1 for further analysis. Then, to analyze the expression of AVL9 in LUAD tissues, we collected 535 LUAD tissues and 59 paired adjacent normal lung tissues from the TCGA database, as well as 226 LUAD tissues and 20 normal control tissues from the Oncomine database. As shown in Fig. 2B and c, AVL9 was highly expressed in LUAD tissues in the TCGA and Oncomine databases (*P* < 0.0001). Importantly, high expression of AVL9 indicated poor outcomes in LUAD patients (*P* = 0.007) (Fig. 2D) and AVL9 could be used as an independent predictive factor of prognosis in patients with LUAD (*P* < 0.01) (Table 2).

**Table 2:** AVL9 can be used as an independent prognostic factor for lung adenocarcinoma based on the TCGA database.

Variables	Univariate analysis			Multivariate analysis		
	*P* value	HR	95% CI	*P* value	HR	95% CI
AVL9 expression (high/low)	0.003*	1.570	1.160–2.124	0.001*	1.668	1.223–2.275
Clinical‐Stage(I + II/III + IV)	0.000*	2.388	1.737–3.282	0.484	1.173	0.750–1.833
Pathologic‐T (T1 + T2/T3 + T4)	0.000*	2.228	1.519–3.266	0.001*	2.035	1.322–3.132
Pathologic‐M (M0/M1)	0.021*	1.948	1.105–3.433	0.209	1.497	0.798–2.810
Pathologic‐N (N0/N1 + N2+N3)	0.000*	2.483	1.841–3.348	0.000*	2.095	1.462–3.001
Age(<60/≥60)	0.752	1.056	0.753–1.480			
Gender (female/male)	0.520	1.103	0.819–1.486			

HR, hazard ratio.

*P < 0.05.

To further confirm the relationship between ALMS1‐IT1 and AVL9, qRT‐PCR analysis and a western blot assay were performed. After Calu‐3 cells were transfected with si‐ALMS1‐IT1, the mRNA and protein levels of AVL9 were both obviously declined compared to the control group. However, this decrease resulting from ALMS1‐IT1 silencing can be reversed by the overexpression of AVL9 (*P* < 0.01) (Fig. 2E,F). Consistently, up‐regulation of ALMS1‐IT1 enhanced the expression of AVL9 in NCI‐H2009 cells at both the mRNA and protein levels, whereas AVL9 expression was clearly decreased after NCI‐H2009 cells were co‐transfected with pcDNA3.1‐ALMS1‐IT1 and si‐AVL9 compared to the pcDNA3.1‐ALMS1‐IT1 group (*P* < 0.01) (Fig. 2G,H). These findings demonstrated that ALMS1‐IT1 controlled AVL9 expression and was positively related to its expression.

**Fig. 3:**
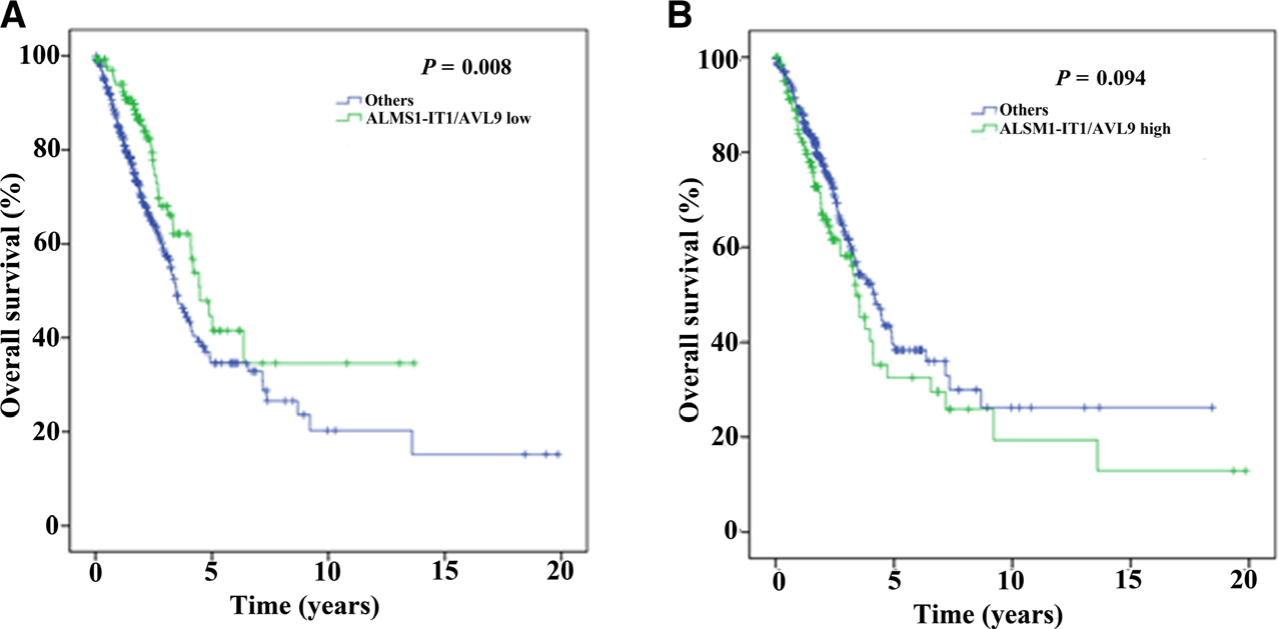
The synergistic effect of ALMS1‐IT1 and AVL9 on survival time. (A) The correlation of low expression of ALMS1‐IT1 and AVL9 with survival (*P* < 0.01). (B) The correlation of high expression of ALMS1‐IT1 and AVL9 with survival (*P* = 0.094).

Additionally, the impact of ALMS1‐IT1 combined with AVL9 on the survival of patients with LUAD was analyzed based on the TCGA database. As shown in Fig. 3, the prognosis of LUAD patients with low expression of both ALMS1‐IT1 and AVL9 was better compared to that of the other patients (*P* = 0.008), although patients with high expression of both ALMS1‐IT1 and AVL9 had no difference in prognosis compared to the other patients (*P* = 0.094).

### The impacts of ALMS1‐IT1/AVL9 on the malignant biological behaviors of LUAD cells

**Fig. 4:**
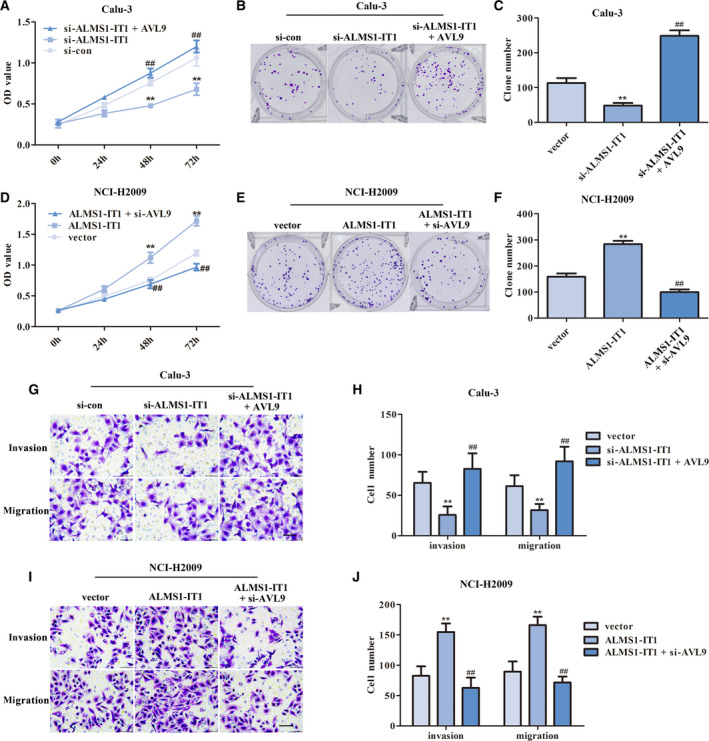
The impact of ALMS1‐IT1/AVL9 on the malignant phenotype of LUAD cells. (A) Cell viability of transfected Calu‐3 cells was monitored using a CCK‐8 assay. **P < 0.01 vs. control group, ##*P* < 0.01 vs. si‐ALMS1‐IT1 group. (B, C) A colony formation assay was carried out to measure the proliferation of transfected Calu‐3 cells. **P < 0.01 vs. control group, ##*P* < 0.01 vs. si‐ALMS1‐IT1 group. (D) Cell viability of transfected NCI‐H2009 cells was monitored using a CCK‐8 assay. **P < 0.01 vs. control group, ##*P* < 0.01 vs. si‐ALMS1‐IT1 group. (E, F) A colony formation assay was carried out to measure the proliferation of transfected NCI‐H2009 cells. **P < 0.01 vs. control group, ##*P* < 0.01 vs. si‐ALMS1‐IT1 group. (G, H) The invasion and migration of transfected Calu‐3 cells were measured using a transwell assay. **P < 0.01 vs. control group, ##*P* < 0.01 vs. si‐ALMS1‐IT1 group. (I, J) The invasion and migration of transfected NCI‐H2009 cells were also measured using a transwell assay. **P < 0.01 vs. control group, ##*P* < 0.01 vs. si‐ALMS1‐IT1 group. Scale bar =200 µm. Error bars represent the mean ± SD derived from three independent experiments. Comparisons between groups were analyzed using *t‐*tests (two‐sided).

To clarify the function of ALMS1‐IT1/AVL9 in the malignant progression of LUAD, we performed functional experiments *in vitro*. First, the knockdown efficiency of si‐ALMS1‐IT1 and si‐AVL9 was detected using a qRT‐PCR. The results are shown in Fig. S1. Then, CCK‐8 and colony formation assays were conducted to assess the proliferation of transfected cells. As shown in Fig. 4A–C, the viability of Calu‐3 cells and the number of colonies were markedly decreased in cells lacking ALMS1‐IT1, whereas the proliferative capacity was clearly enhanced in Calu‐3 cells co‐transfected with si‐ALMS1‐IT1 and pcDNA3.1‐AVL9 compared to the si‐ALMS1‐IT1 group (*P* < 0.01). As expected, overexpression of ALMS1‐IT1 promoted cell viability and heightened the number of colonies in NCI‐H2009 cells, whereas down‐regulation of AVL9 almost cancelled the ALMS1‐IT1 overexpression‐induced promotion of proliferation (*P* < 0.01) (Fig. 4D–F).

Subsequently, a transwell assay was performed to measure the influence of ALMS1‐IT1/AVL9 on the invasion and migration of LUAD cells. The results revealed that the number of cells invading and migrating in Calu‐3 cells transfected with si‐ALMS1‐IT1 was obviously decreased compared to the control, whereas up‐regulation of AVL9 can reverse the si‐ALMS1‐IT1‐mediated suppression with respect to invasion and migration of Calu‐3 cells (*P* < 0.01) (Fig. 4G,H). By contrast, after transfection with pcDNA3.1‐ALMS1‐IT1, the invasion and migration of NCI‐H2009 cells showed a notable increase. Nevertheless, the increase resulting from overexpression of ALMS1‐IT1 can be hindered via knockdown of AVL9 in NCI‐H2009 cells (*P* < 0.01) (Fig. 4I,J). Our findings indicated that ALMS1‐IT1 modulated the malignant phenotype of LUAD cells at least partially by regulating AVL9, thus affecting the malignant progression of LUAD.

### The impact of ALMS1‐IT1/AVL9 on the function of LUAD cells may be related to the cyclin‐dependent kinase pathway

**Fig. 5:**
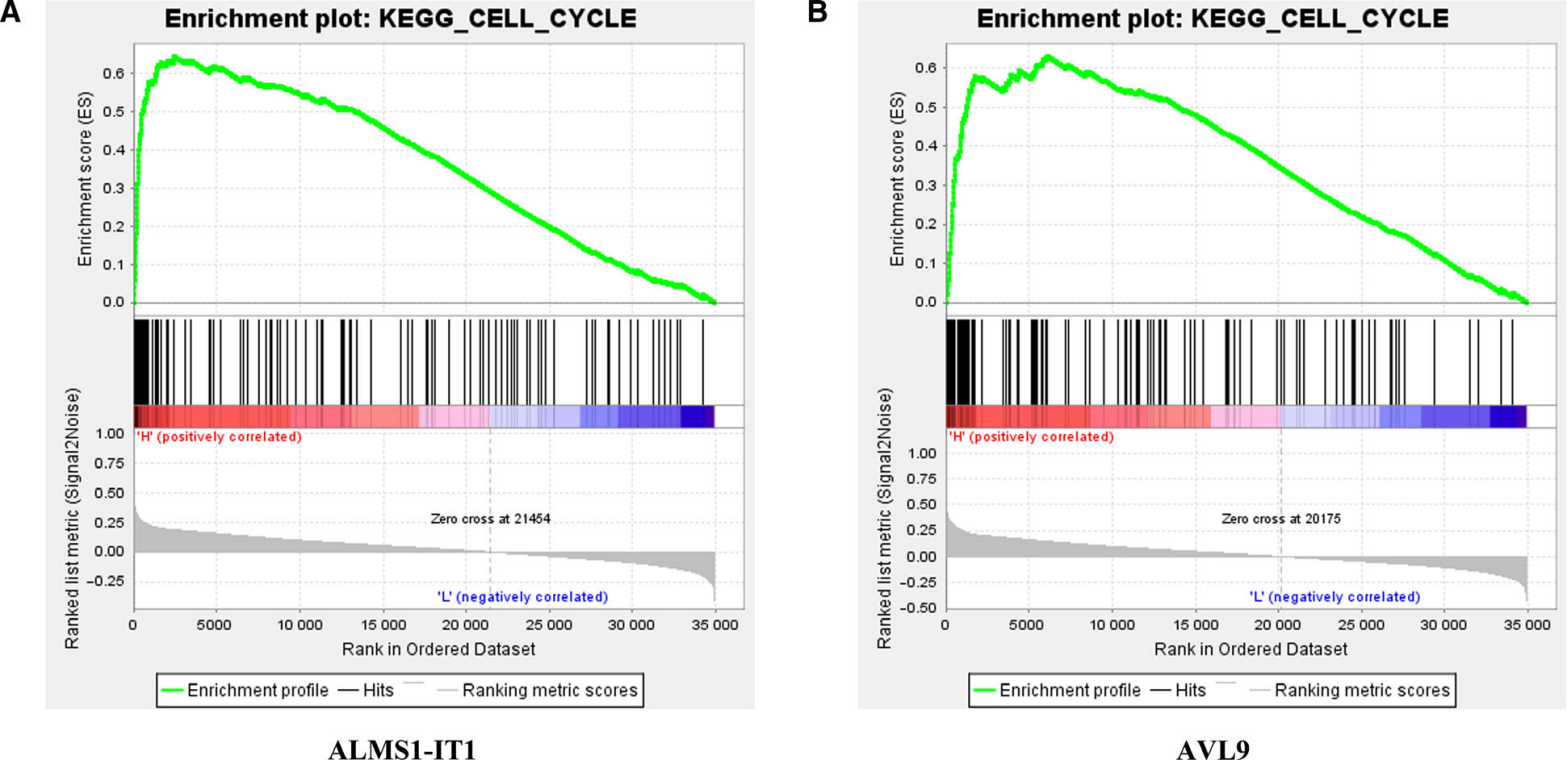
GSEA analysis was used to analyze the pathways closely related to the high expression of (A) ALMS1‐IT1 and (B) AVL9.

**Fig. 6:**
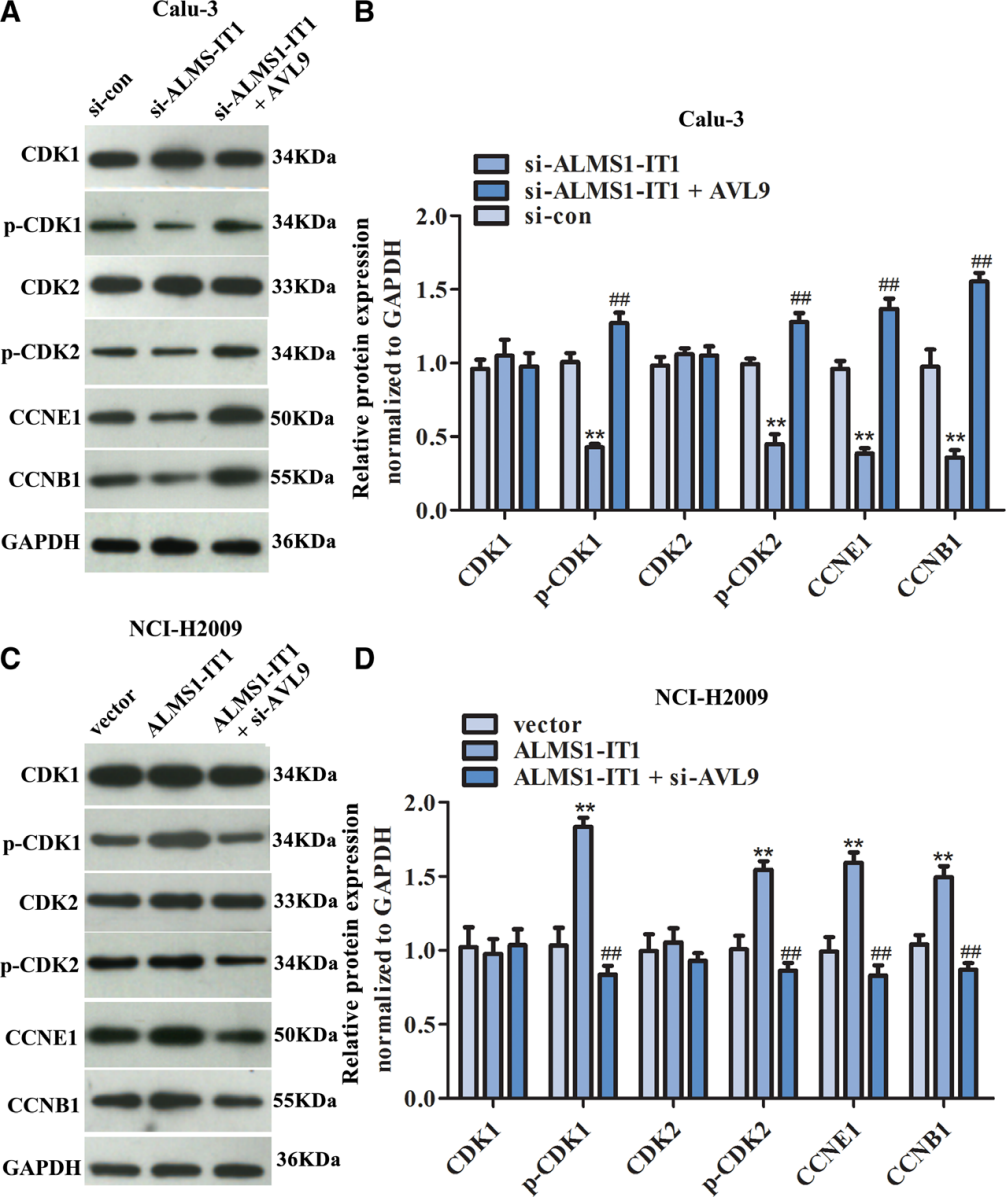
The impact of ALMS1‐IT1/AVL9 on the CDK pathway of LUAD cells. (A, B) CDK‐related proteins in Calu‐3 cells were detected using a western blot assay. **P < 0.01 vs. control group, ##*P* < 0.01 vs. si‐ALMS1‐IT1 group. (C, D) CDK‐related proteins in NCI‐H2009 cells were detected using a western blot assay. **P < 0.01 vs. control group, ##*P* < 0.01 vs. si‐ALMS1‐IT1 group. Error bars represent the mean ± SD derived from three independent experiments. Comparisons between groups were analyzed using *t‐*tests (two‐sided).

To further investigate the underlying mechanism of ALMS1‐IT1/AVL9 in LUAD, we analyzed the pathway related to ALMS1‐IT1/AVL9 using GSEA analysis. From Fig. 5, it can be seen that the cell cycle pathway was closely related to the high expression of both ALMS1‐IT1 and AVL9 in LUAD. Because the cell cycle is regulated by cyclin‐dependent kinase (CDKs) and related regulatory factors [22], a western bolt assay was performed to measure the expression of CDKs‐related proteins in LUAD. As shown in Fig. 6A,B, the phosphorylation levels of CDK1 and CDK2 and the expression of CCNE1 and CCNB1 in Calu‐3 cells with ALMS1‐IT1 deletion were significantly decreased compared to those in si‐con group, although the expression of total CDK1 and CDK2 was almost unaltered. Assuredly, up‐regulation of AVL9 can reverse the ALMS1‐IT1 suppression‐induced inhibitory effect on the CDK pathway in Calu‐3 cells. On the other hand, the phosphorylation levels of CDK1 and CDK2 and the expression of CCNE1 and CCNB1 were clearly enhanced in NCI‐H2009 cells transfected with pcDNA3.1‐ALMS1‐IT1, whereas NCI‐H2009 cells co‐transfected with pcDNA3.1‐ALMS1‐IT1 and si‐AVL9 demonstrated a conspicuous decrease compared to the pcDNA3.1‐ALMS1‐IT1 group (Fig. 6C,D). These results indicate that ALMS1‐IT1/AVL9 may functionally participate in regulating the progression of LUAD via the CDK pathway.

## Discussion

Previously, extensive studies have shown that LUAD, as a type of non‐small cell carcinoma, comprises a typical tumor that can benefit from targeted molecular therapies [23, 24, 25]. In the present study, we found for the first time that the expression of ALMS1‐IT1 and AVL9 was both markedly elevated in LUAD, and there was a co‐expression relationship between ALMS1‐IT1 and AVL9. Also, the prognosis of LUAD patients with low expression of ALMS1‐IT1 and AVL9 was better compared to that of other patients. Furthermore, biological functional experiments *in vitro* revealed that ALMS1‐IT1/AVL9 can modulate the malignant biological behaviors of LUAD cells, which may be realized via the CDK pathway.

Recently, ALMS1‐IT1 has been considered as a key gene in small‐cell lung cancer and may be a potential biomarker for its occurrence [26]. Our data confirmed that ALMS1‐IT1 was highly expressed in LUAD, and high ALMS1‐IT1 expression caused poor outcomes in patients with LUAD. Importantly, overexpression of ALMS1‐IT1 contributes to promoting the viability of LUAD cells *in vitro*. These findings indicated that the level of ALMS1‐IT1 was related to the malignant transformation of LUAD cells and it may be a novel biomarker for LUAD therapy. So far, the specific functional mechanism of lncRNAs has not been fully elucidated [27] and so we further investigated how ALMS1‐IT1 functions in LUAD. Bioinformatics analysis revealed that AVL9 is a co‐expression mRNA of ALMS1‐IT1, which was positively correlated with ALMS1‐IT1 expression in LUAD. As an oncogene [17], AVL9 has been investigated in multiple cancers. To our knowledge, AVL9 is a momentous transporter for promoting cell migration [21]. For example, high expression of AVL9 in clear cell renal cell carcinoma accelerates cell migration [28]. Furthermore, in patients with gastric cancer, the high expression of AVL9 leads to poor prognosis [29]. In the present study, we confirmed that AVL9 was up‐regulated in LUAD and could be used as an independent predictive factor of prognosis in patients with LUAD. Furthermore, LUAD patients with low expression of ALMS1‐IT1 and AVL9 had a better survival rate compared to the other patients. For the first time, we have revealed that ALMS1‐IT1/AVL9 was involved in the modulation of the malignant phenotype of LUAD cells. These data indicated that ALMS1‐IT1 promoted the malignant progression of LUAD at least partially by regulating AVL9 expression and ALMS1‐IT1/AVL9 showed a synergistic effect in LUAD.

The abnormal changes and regulation of the cell cycle are closely related to the carcinogenesis of cells [30, 31] and its disorder inevitably occurs in various types of tumors. Previous studies have shown that the cell cycle is regulated by CDKs, as well as by related regulatory factors [22, 32]. Briefly, the suppression of CDKs can result in cell cycle arrest and apoptosis, thus inhibiting the progression of cancer [22]. Gopinathan *et al*. [33] reported that the absence of CDK2 and cyclin A2 can inhibit the proliferation of mouse embryonic fibroblasts and delay the occurrence of tumor. Cyclin E1 (CCNE1) is an important factor that regulates the proliferation of lung cancer cells into the S phase and G1 phase, which is reported to function in regulating the growth of lung cancer cells [34]. In the present study, KEGG analysis revealed that cell cycle pathway was closely related to the both high expression of ALMS1‐IT1 and AVL9 in LUAD. Moreover, AVL9 has been reported to play an important role in cell cycle progression [17, 35]. Hence, we investigated the effects of ALMS1‐IT1 and AVL9 on the CDK pathway. As a result, ALMS1‐IT1/AVL9 can regulate the phosphorylation levels of CDK1 and CDK2, as well as the expression of CCNE1 and CCNB1. Based on this, we speculate that ALMS1‐IT1 regulates AVL9 by adsorbing miRNA and thus participates in the regulation of cell cycle‐related CDK pathway. However, the specific mechanism, either direct regulation or indirect regulation, needs further study.

There are some limitations to the present study, such that the potential mechanisms how ALMS1‐IT1 may regulate AVL9 expression needs to be further elucidated. Indeed, although we have begun to investigate the mechanism of ALMS1‐IT1 with respect to regulating AVL9, this is still in progress. Various lncRNAs can regulate the expression of mRNAs by acting as competing endogenous RNAs or miRNA sponges. Based on bioinformatics prediction, we obtained three down‐regulated miRNAs that can be sponged by ALMS1‐IT1 and can also target AVL9 mRNA, namely hsa‐miR‐206, hsa‐miR‐4524a and hsa‐miR‐6504 (Fig. S2a,b). The survival curve revealed that patients with high expression of hsa‐miR‐206 have a good prognosis, whereas the expression of hsa‐miR‐4524a and hsa‐miR‐6504 has no correlation with the prognosis (Fig. 2C–E). Moreover, we analyzed the interaction between these three miRNAs and ALMS1‐IT1/AVL9. As shown in Fig. S2f–h, the expression of ALMS1‐IT1 is negatively correlated with the expression of miR‐4524a but has no correlation with the expression of miR‐206 and miR‐6504. Also, the expression of AVL9 was negatively correlated with the expression of miR‐206 and miR‐4524a but has no correlation with the expression of miR‐6504 (Fig. S2i–k). These represent the results of our current research, although the specific mechanism and biological function needs to be investigated further.

In summary, ALMS1‐IT1/AVL9 showed a synergistic effect in regulating the progression of LUAD. Our findings have demonstrated that ALMS1‐IT1/AVL9 was involved in modulating the malignant progression of LUAD *in vitro*, and the regulatory impact was at least partially achieved through the CDK pathway. This provides a novel strategy for the targeted molecular therapy of LUAD, and an animal experimental model will be established in the future.

## Acknowledgements

This study funded by National Cancer Center fund project (NCC201808B015).

## Conflict of interest

The authors declare that they have no conflict of interest.

## Author contributions

LT, ZTY and HSL designed the study and collected data. LT, LZH and GBX analyzed the data. LT, ZTY, XJY and LJ wrote the manuscript. LT, LMX and HSL reviewed and edited the manuscript. All authors read and approved the final manuscript submitted for publication.

## Supporting information


**Fig. S1.** The knockdown efficiency of si‐ALMS1‐IT1and si‐AVL9 was detected using qRT‐PCR. **P < 0.01. Error bar represents the mean ± SD derived from three independent experiments. Comparisons between groups were analyzed using *t*‐tests (two‐sided).

**Fig. S2.** The mechanism of ALMS1‐IT1 regulating AVL9 was predicted using bioinformatics prediction. (a) A Wayne diagram was applied to determine the possible targets of ALMS1‐IT1. (b) The expression of hsa‐miR‐206, hsa‐miR‐4524a and hsa‐miR‐6504 in LUAD based on the TCGA database. *P* < 0.01. (c‐e) Kaplan–Meier analysis was used to analyze the relevance of hsa‐miR‐6504 (*P* = 0.42), hsa‐miR‐206 (*P* = 0.03) and hsa‐miR‐4524a (*P* = 0.15) expression and survival rate in LUAD patients. (f, g) Pearson’s correlation coefficient was utilized to analyze the correlation between ALMS1‐IT1 and hsa‐miR‐206 (*r* = –0.0461, *P* = 0.36), hsa‐miR‐4524a (*r* = –0.1292, *P* = 0.01) and hsa‐miR‐6504 (*r* = 0.002, *P* = 0.97). (i–k) Pearson’s correlation coefficient was utilized to analyze the correlation between AVL9 and hsa‐miR‐206 (*r* = –0.1629, *P* = 0.001), hsa‐miR‐4524a (*r* = –0.1102, *P* = 0.03) and hsa‐miR‐6504 (*r* = 0.01, *P* = 0.86).

## Data Availability

The data that support the findings of this study are available from the corresponding author upon reasonable request.
