# Supplementary figures and images for: The lncRNA ALMS1‐IT1 may promote malignant progression of lung adenocarcinoma via AVL9‐mediated activation of the cyclin‐dependent kinase pathway
Source: FEBS Open Bio. 2021 Apr 3;11(5):1504–15. doi: 10.1002/2211-5463.13140 (PMC8091588; doi:10.1002/2211-5463.13140)

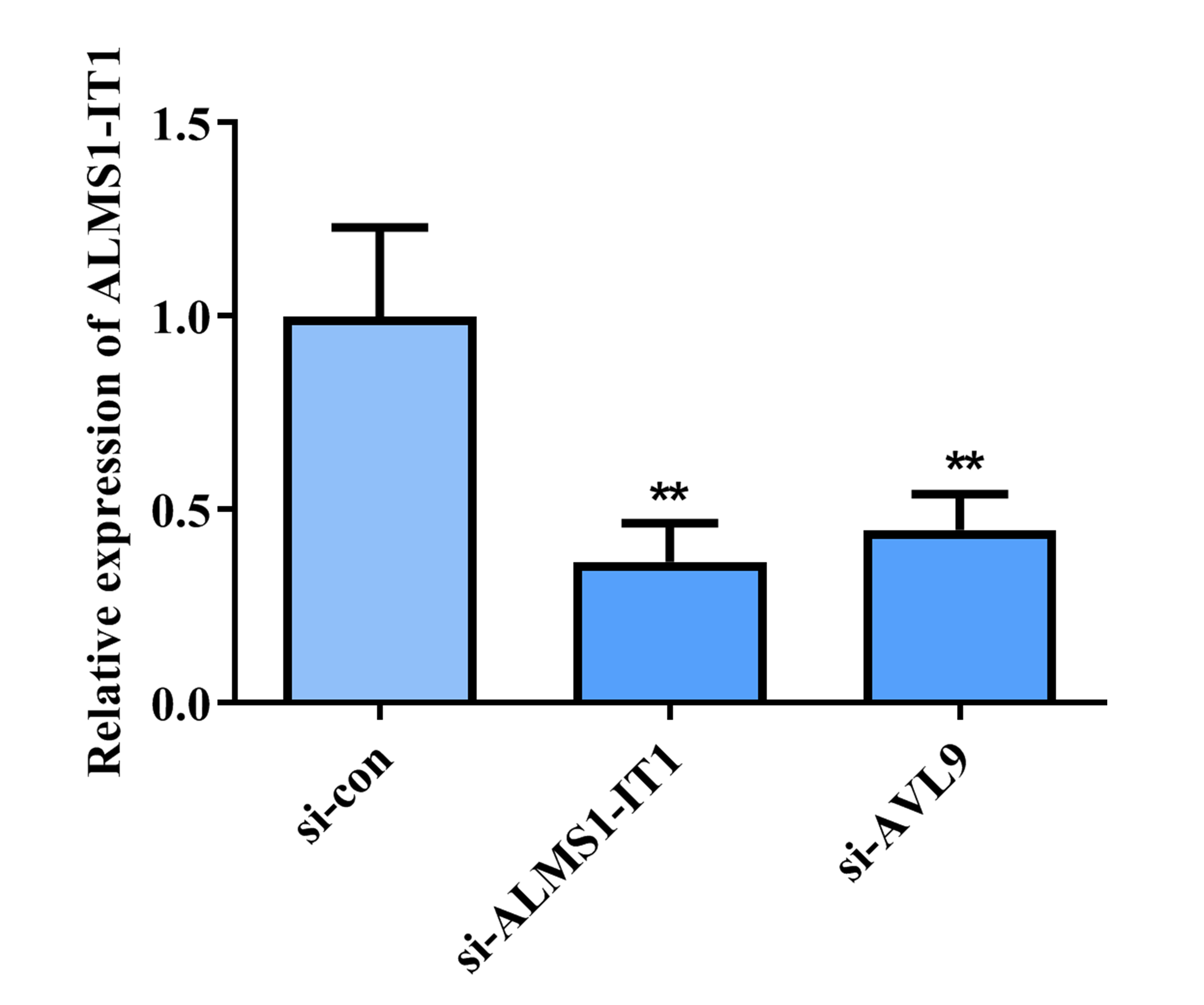

Supplement: Supplementary file 1 — Fig. S1. The knockdown efficiency of si‐ALMS1‐IT1and si‐AVL9 was detected using qRT‐PCR. **P < 0.01. Error bar represents the mean ± SD derived from three independent experiments. Comparisons between groups were analyzed using t‐tests (two‐sided). [file FEB4-11-1504-s002.tif]

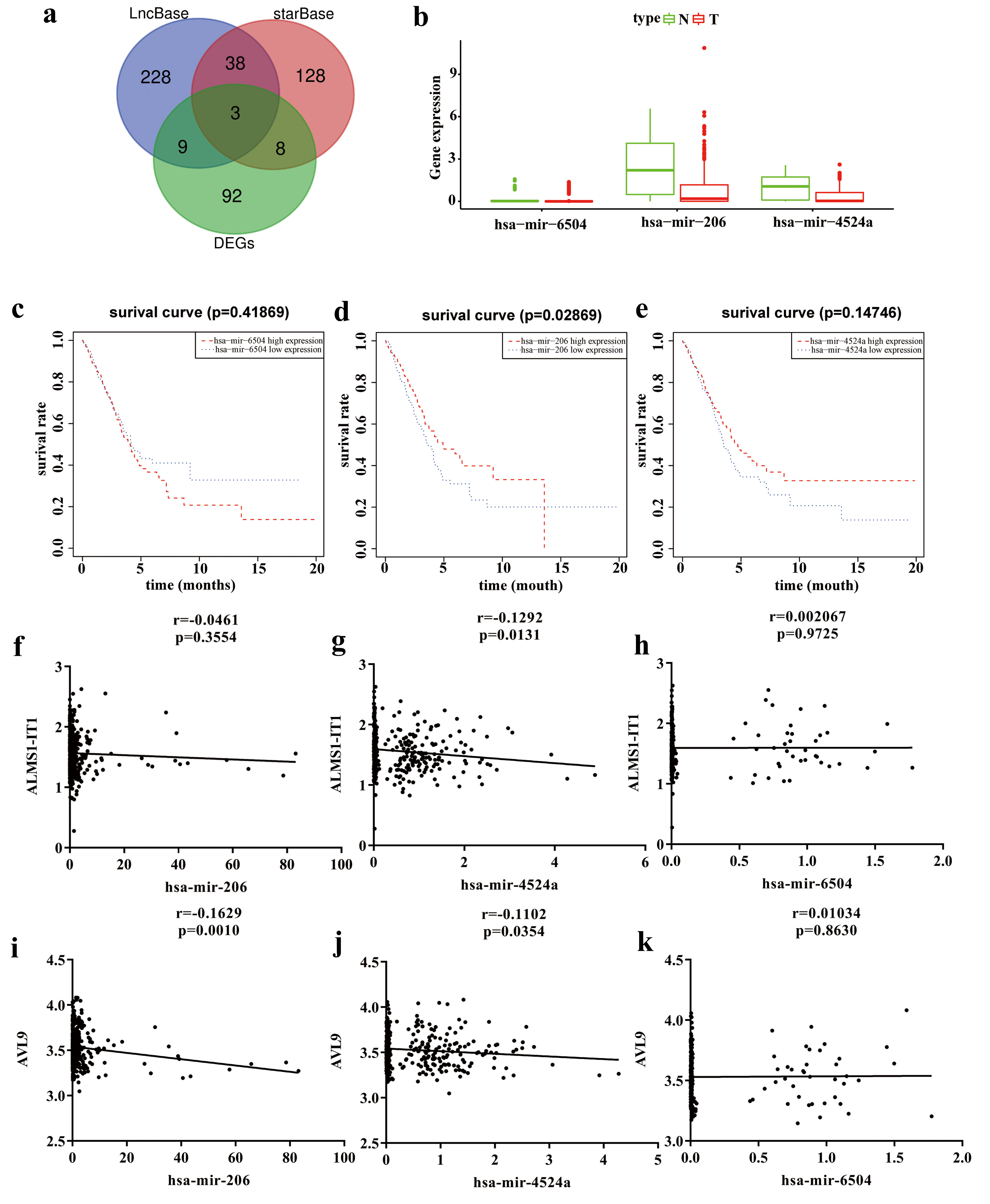

Supplement: Supplementary file 2 — Fig. S2. The mechanism of ALMS1‐IT1 regulating AVL9 was predicted using bioinformatics prediction. (a) A Wayne diagram was applied to determine the possible targets of ALMS1‐IT1. (b) The expression of hsa‐miR‐206, hsa‐miR‐4524a and hsa‐miR‐6504 in LUAD based on the TCGA database. P < 0.01. (c‐e) Kaplan–Meier analysis was used to analyze the relevance of hsa‐miR‐6504 (P = 0.42), hsa‐miR‐206 (P = 0.03) and hsa‐miR‐4524a (P = 0.15) expression and survival rate in LUAD patients. (f, g) Pearson’s correlation coefficient was utilized to analyze the correlation between ALMS1‐IT1 and hsa‐miR‐206 (r = –0.0461, P = 0.36), hsa‐miR‐4524a (r = –0.1292, P = 0.01) and hsa‐miR‐6504 (r = 0.002, P = 0.97). (i–k) Pearson’s correlation coefficient was utilized to analyze the correlation between AVL9 and hsa‐miR‐206 (r = –0.1629, P = 0.001), hsa‐miR‐4524a (r = –0.1102, P = 0.03) and hsa‐miR‐6504 (r = 0.01, P = 0.86). [file FEB4-11-1504-s001.jpg]
